# Dysfunctional mitochondria, disrupted levels of reactive oxygen species, and autophagy in B cells from common variable immunodeficiency patients

**DOI:** 10.3389/fimmu.2024.1362995

**Published:** 2024-03-26

**Authors:** Maria Berman-Riu, Vanesa Cunill, Antonio Clemente, Antonio López-Gómez, Jaime Pons, Joana M. Ferrer

**Affiliations:** ^1^ Department of Immunology, Son Espases University Hospital, Palma, Spain; ^2^ Human Immunopathology Research Laboratory, Health Research Institute of the Balearic Islands (IdISBa), Palma, Spain; ^3^ Multidisciplinary Sepsis Group, Health Research Institute of the Balearic Islands (IdISBa), Palma, Spain; ^4^ CIBER de Enfermedades Infecciosas (CIBERINFEC), Instituto de Salud Carlos III, Madrid, Spain; ^5^ CIBER de Enfermedades Respiratorias (CIBERES), Instituto de Salud Carlos III, Madrid, Spain

**Keywords:** B cells, CVID, autophagy, ROS, mitochondria

## Abstract

**Introduction:**

Common Variable Immunodeficiency (CVID) patients are characterized by hypogammaglobulinemia and poor response to vaccination due to deficient generation of memory and antibody-secreting B cells. B lymphocytes are essential for the development of humoral immune responses, and mitochondrial function, hreactive oxygen species (ROS) production and autophagy are crucial for determining B-cell fate. However, the role of those basic cell functions in the differentiation of human B cells remains poorly investigated.

**Methods:**

We used flow cytometry to evaluate mitochondrial function, ROS production and autophagy processes in human naïve and memory B-cell subpopulations in unstimulated and stimulated PBMCs cultures. We aimed to determine whether any alterations in these processes could impact B-cell fate and contribute to the lack of B-cell differentiation observed in CVID patients.

**Results:**

We described that naïve CD19^+^CD27^-^ and memory CD19^+^CD27^+^ B cells subpopulations from healthy controls differ in terms of their dependence on these processes for their homeostasis, and demonstrated that different stimuli exert a preferential cell type dependent effect. The evaluation of mitochondrial function, ROS production and autophagy in naïve and memory B cells from CVID patients disclosed subpopulation specific alterations. Dysfunctional mitochondria and autophagy were more prominent in unstimulated CVID CD19^+^CD27^-^ and CD19^+^CD27^+^ B cells than in their healthy counterparts. Although naïve CD19^+^CD27^-^ B cells from CVID patients had higher basal ROS levels than controls, their ROS increase after stimulation was lower, suggesting a disruption in ROS homeostasis. On the other hand, memory CD19^+^CD27^+^ B cells from CVID patients had both lower ROS basal levels and a diminished ROS production after stimulation with anti-B cell receptor (BCR) and IL-21.

**Conclusion:**

The failure in ROS cell signalling could impair CVID naïve B cell activation and differentiation to memory B cells. Decreased levels of ROS in CVID memory CD19^+^CD27^+^ B cells, which negatively correlate with their *in vitro* cell death and autophagy, could be detrimental and lead to their previously demonstrated premature death. The final consequence would be the failure to generate a functional B cell compartment in CVID patients.

## 1 Introduction

Common Variable Immunodeficiency (CVID) is the commonest symptomatic primary humoral immunodeficiency, characterized by hypogammaglobulinemia and poor response to vaccination (1). Patients benefit from substitutive gammaglobulin therapy. Apart from recurrent infections, some patients present with non-infectious complications including autoimmune, autoinflammatory, and lymphoproliferative disorders requiring immunosuppressive or other treatments different from gammaglobulin (2–4). Monogenic mutations in genes related to inborn errors of immunity (IEI) have been described in approximately 30% of the patients (5).

The common finding of abnormal late B-cell differentiation to memory and antibody-secreting cells (ASCs) found in CVID patients has provided the basis for several classifications that rely on the presence or absence of different subpopulations of memory B cells (6–8). The generation of memory B cells and ASC is essential for the development of humoral immune responses. For this to occur, mature B lymphocytes must receive signals provided by antigens through B-cell receptor (BCR) and T-cell help. T-cell cooperation is established through direct contact between T-cell membrane molecules and their corresponding B-cell ligands or through the secretion of cytokines that stimulate receptors located on the B-cell surface. One of the most important cytokines for B-cell differentiation is IL-21. In certain circumstances, stimulation through toll-like receptor (TLR) can substitute for T-cell help, driving B-cell differentiation (9–11). These stimuli also influence the apoptosis/survival balance that preserves B-cell homeostasis; specific requirements have been shown to be dependent on the B-cell maturation and activation status (12, 13).

Immune cell metabolism provides energy and a source of biomolecules but also determines immune-cell fate. Mitochondrial metabolic pathways play essential roles in cell activity, differentiation, stress, and aging and are important for B-cell activation and plasma cell generation (14–17). Oxidative stress is associated with the intracellular production of reactive oxygen species (ROS), mainly generated in the inner membrane of the mitochondria by the electron transport chain (18). Although ROS play important roles in the regulation of cell signaling and homeostasis (19–21), excessive ROS can lead to cellular damage by oxidation of proteins, lipids, nucleic acids, and organelles. Impairment of mitochondrial function leads to depolarization, reduction in membrane potential, changes in membrane permeability, and, ultimately, apoptosis (22).

Autophagy, an evolutionary conserved lysosomal degradation process, protects cells from oxidative stress by removing damaged cellular components such as mitochondria (23, 24). Its role in memory and plasma B-cell development has been assessed only in mouse models (25). There are lines of evidence suggesting a reciprocal interplay between mitochondrial function and autophagy, but the molecular mechanisms underlying autophagy response to oxidative stress are largely unknown (26).

We have previously found that an increased susceptibility to activation-induced apoptosis could be the cause of memory B-cell loss in a subgroup of CVID patients with a more compromised memory B-cell compartment (27). We have also demonstrated a disbalance in mitochondrial apoptosis regulation in this subgroup of patients (28).

The aim of this study was to examine the interplay of mitochondrial function, ROS production, and autophagy processes in healthy human naïve and memory B-cell subpopulations and to determine whether any alterations in these processes could potentially impact B-cell fate and contribute to the lack of B-cell differentiation observed in CVID patients.

## 2 Materials and methods

### 2.1 Patients

CVID patients were selected according to diagnostic criteria established by the International Union of Immunological Societies scientific group for primary immunodeficiency diseases (29, 30). The study included 25 patients diagnosed with CVID, comprising 17 women and eight men, with ages ranging from 31 to 78 and 18 to 74, respectively. Only patients with more than 1% of B cells were included in the study. Patients were categorized into two groups according to the European consensus classification for CVID (EUROclass) (8): i) CVID patients with <2% of IgD^−^CD27^+^ (switched memory phenotype) B cells or smB− (n = 13) and (ii) patients with >2% of IgD^−^CD27^+^ (n = 12) or smB+. All patients received substitutive gammaglobulin therapy every 21–28 days and were free from infection at the time of the study. Peripheral blood samples were collected before gammaglobulin replacement.

**Table 1** provides a summary of the patients’ age, gender, and percentages of B-cell populations. Additionally, 25 age and sex-matched healthy blood donors were included as controls (**Supplementary Table 1**). The study was conducted in accordance with the ethical principles outlined in the 1975 Declaration of Helsinki, and it was approved by CEIC-IB (Balearic Islands Clinical Research Ethics Committee; IB 4322/20). Written informed consent was obtained from all subjects prior to their participation in the study.

**Table 1:** Age, gender, immunoglobulin levels, B-cell subpopulations, and classification of the CVID patient cohort.

Patient	Current age (years)	Gender (male/female)	IgG (mg/dL)	IgA (mg/dL)	IgM (mg/dL)	CD19^+^ (%)	CD19^+^ CD21^low^ (%)	CD19^+^ IgD^+^CD27^−^ (%)	CD19^+^ IgD^+^CD27^+^ (%)	CD19^+^ IgD^−^CD27^+^ (%)	EUROclass group
1	49	M	85	<24	<18	3	16	73	25	<1	smB−
2	71	F	112	<6	<5	6	16	87	7	<1	smB−
3	55	F	288	32	14	20	NA	83	3	3	smB+
4	51	F	387	<7	17	6	NA	67	15	9	smB+
5	72	F	327	73	30	16	7	62	27	8	smB+
6	44	F	578	<25	62	11	20	61	36	3	smB+
7	40	F	164	<7	8	24	5	89	6	<2	smB−
8	41	F	351	20	<6	15	24	81	11	<2	smB−
9	58	F	495	44	38	11	6	72	35	2	smB+
10	73	M	316	9	12	9	36	94	1	<1	smB−
11	78	F	452	46	58	6	3	14	26	56	smB+
12	41	M	82	<6	5	11	11	93	6	1	smB−
13	52	M	464	<6	48	8	41	66	27	3	smB+
14	60	F	397	126	22	20	3	90	9	1	smB−
15	33	F	172	<6	11	5	15	68	25	7	smB+
16	55	M	117	31	30	5	19	74	25	1	smB−
17	45	F	371	<6	34	5	35	80	19	1	smB−
18	74	M	452	40	30	4	69	93	3	<1	smB−
19	78	F	434	47	46	12	11	32	54	10	smB+
20	44	F	200	<7	6	6	57	87	9	<1	smB+
21	36	M	13	<7	<5	4	5	96	3	<1	smB−
22	54	F	548	<7	<5	7	43	90	7	1	smB−
23	42	F	465	<7	97	17	18	68	28	3	smB+
24	31	F	450	<7	73	10	18	60	33	5	smB+
25	18	M	306	<5	<5	2	27	94	3	<1	smB−

Current age (years), gender (M, male; F, female), serum immunoglobulin levels (IgG, IgA, and IgM) before starting replacement therapy, percentage of peripheral blood B cells (CD19^+^), percentages of CD21^low^, naïve (IgD^+^CD27^−^), unswitched memory (IgD^+^CD27^+^), and switched memory (IgD^−^CD27^+^) B-cell subpopulations (referred to total CD19^+^ B cells), EUROclass classification.

NA, not available.

### 2.2 Cell culture

Peripheral blood mononuclear cells (PBMCs) were isolated from heparinized blood by density gradient centrifugation (Lymphoprep™; StemCell, Technologies Inc., Vancouver, BC, Canada). PBMCs were resuspended in culture medium: RPMI-1640 formulated with glutamine (300 mg/L) (Irvine Scientific, Santa Ana, CA, USA) and supplemented with 10% heat-inactivated fetal calf serum (Cytiva, Marlborough, MA, USA) and antibiotics (penicillin and streptomycin) (EuroClone, Milan, Italy).

PBMCs were cultured (1 × 10^6^ cell/mL) in 24-well flat bottom plates (for ROS and autophagy evaluation) or 96-well plates (for mitochondrial evaluation) and incubated in the absence (unstimulated) or presence of specific B-cell stimulus combinations: F(ab)2 goat anti-human IgA + IgG + IgM (anti-BCR) (5 µg/mL; Jackson ImmunoResearch, West Grove, PA, USA), anti-human CD40/TNFRSF5 antibody (1 µg/mL; R&D Systems, Minneapolis, MN, USA), CpG-oligodeoxinucleotide (ODN) (0.6 µg/mL; CpG-ODN 2006 type B; InvivoGen, San Diego, CA, USA), and human recombinant IL-21 (100 ng/mL; BioSource, Blue Bell, PA, USA). Additionally, in cultures aimed at autophagic flux evaluation, bafilomycin A1 (10 nM; Sigma-Aldrich, St. Louis, MO, USA) was added to inhibit autophagosome degradation. Cultures were maintained for 24 h at 37°C in a 5% CO_2_ atmosphere.

### 2.3 Flow cytometry

Peripheral blood lymphocyte populations, cell death, mitochondrial membrane potential (MMP), mitochondrial mass (MM), ROS production, and autophagy levels were analyzed by flow cytometry using a BD FACSLyric (Becton Dickinson, Franklin Lakes, NJ, USA) cytometer and FlowJo v10 software for data analysis.

#### 2.3.1 Peripheral blood lymphocyte populations

A surface staining protocol was applied to evaluate B-cell subpopulations and phenotypically classify CVID patients. Briefly, 50 µL of peripheral whole blood was incubated with a combination of fluorochrome-conjugated monoclonal antibodies (5 µL of each antibody) for 20 min at room temperature (25°C). Red blood cells were lysed for 10 min with 2 mL of FACS Lysing solution (Becton Dickinson) and washed with phosphate-buffered saline (PBS) before flow cytometry analysis. The combination of the following monoclonal antibodies was used to determine the distribution of B-cell subpopulations—anti-CD19-PECy7, anti-CD45-V500, anti-CD27-APC, anti-IgD-V450, and anti-CD21-BV605—all from Becton Dickinson.

#### 2.3.2 Mitochondrial function

Mitochondrial function was assessed using MitoTracker probes (MitoTracker Green and MitoTracker Deep Red from Invitrogen, Carlsbad, CA, USA) following the manufacturer’s instructions. Briefly, 2 × 10^5^ harvested cells were stained with Live/Dead Aqua Dead Cell Stain (Invitrogen Molecular Probes, Eugene, OR, USA) for 20 min at room temperature (RT) followed by anti-CD19-PE-Cy7 and anti-CD27-BV421 (both from BD Biosciences, San Jose, CA, USA) staining for 15 min at RT. Then, cells were washed with PBS and stained with MitoTracker Green and MitoTracker Deep Red for the evaluation of mitochondrial mass and mitochondrial membrane potential, respectively, for 30 min at 37°C.

**Figure 1:**
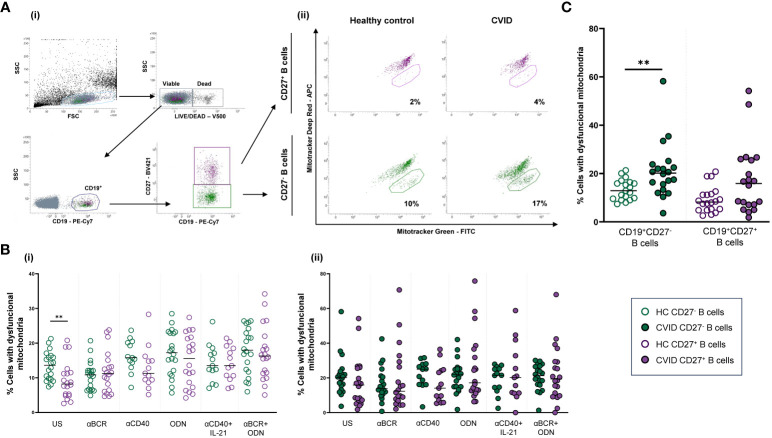
B-cell subpopulations with dysfunctional mitochondria in healthy controls and common variable immunodeficiency (CVID) patients. **(A)** Dot plots of B-cell subpopulations with dysfunctional mitochondria from a representative control and CVID patient. (i) Gating strategy: viable B cells were selected based on forward and side scatter, non-expression of Live/Dead marker, and CD19^+^ expression. B-cell subpopulations were identified as naïve CD19^+^CD27^−^ (green color) and memory CD19^+^CD27^+^ (purple color). (ii) Cells with dysfunctional mitochondria were selected as MitoTracker Deep Red^low^ and MitoTracker Green^+^ from healthy control (left panels) and CVID patients (right panels). **(B)** Comparison between the percentages of naïve CD19^+^CD27^−^ (green dots) and memory CD19^+^CD27^+^ (purple dots) B cells with dysfunctional mitochondria in (i) healthy controls and (ii) CVID patients after 24 h of culture without or with stimulation with anti-BCR, anti-CD40, CpG-ODN, or the combinations anti-CD40+IL-21 and anti-BCR+CpG-ODN. **(C)** Percentages of unstimulated naïve CD19^+^CD27^−^ (green dots) and memory CD19^+^CD27^+^ (purple dots) B cells with dysfunctional mitochondria from healthy controls (empty dots) and CVID patients (filled dots). **(B, C)** Each dot represents an individual; black horizontal lines illustrate the median of the group. Mann–Whitney test p*-*values: **p < 0.01.

Reduction of mitochondrial membrane potential is a hallmark of mitochondrial dysfunction. For this reason, viable (Live/Dead^−^) naïve (CD19^+^CD27^−^) and memory (CD19^+^CD27^+^) B cells (**Figure 1A**i), containing mitochondria with low membrane potential, identified as MitoTracker Deep Red^low^ and MitoTracker Green^+^ (**Figure 1A**ii), were considered B cells with dysfunctional mitochondria (CDM). Fold increase in the percentage of CDM induced by each single stimulus related to the unstimulated sample was expressed as a ratio: (single stimulus % CDM)/(unstimulated % CDM).

#### 2.3.3 ROS production

**Figure 2:**
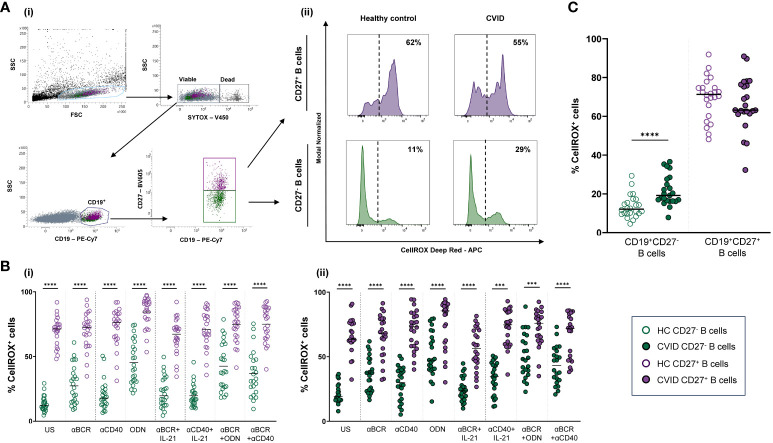
Reactive oxygen species (ROS) in B-cell subpopulations from healthy controls and common variable immunodeficiency (CVID) patients. **(A)** Dot plots with histograms representing ROS production in B-cell subpopulations from a representative control and CVID patient. (i) Gating strategy: viable B cells were selected based on forward and side scatter, non-expression of SYTOX marker, and CD19^+^ expression. B-cell subpopulations were identified as naïve CD19^+^CD27^−^ (green color) and memory CD19^+^CD27^+^ (purple color). (ii) ROS production was identified as the percentage of cells positive for CellROX Deep Red probe (% CellROX^+^ cells). Histograms show the percentage (upper right corner) of ROS-producing cells from healthy control (left histograms) and CVID patients (right histograms). **(B)** Comparison between the percentages of CellROX^+^ naïve CD19^+^CD27^−^ (green dots) and memory CD19^+^CD27^+^ (purple dots) B cells from (i) healthy controls and (i) CVID patients after 24 h of culture without or with stimulation with anti-BCR, anti-CD40, CpG-ODN, anti-BCR+IL-21, anti-CD40+IL-21, anti-BCR+CpG-ODN, or anti-BCR+anti-CD40. **(C)** Percentages of unstimulated CellROX^+^ naïve CD19^+^CD27^−^ (green dots) and memory CD19^+^CD27^+^ (purple dots) from healthy controls (empty dots) and CVID patients (filled dots). **(B, C)** Each dot represents an individual; black horizontal lines illustrate the median of the group. Mann–Whitney test p*-*values: ****p < 0.0001.

CellROX Deep Red Flow Cytometry Assay Kit (Invitrogen Molecular Probes) was used to evaluate ROS production. Briefly, 5 × 10^5^ harvested cells were stained with CellROX Deep Red Reagent (1 µM) for 25 min at 37°C. For surface staining, cells were stained with anti-CD19-PE-Cy7 and anti-CD27-BV605 (all from BD Biosciences) for 15 min at 37°C. Finally, cells were stained with SYTOX Blue Dead Cell Stain (200 nM) for 15 min at 37°C to identify dead (SYTOX^+^) and viable (SYTOX^−^) cells. ROS production was evaluated as the percentage of viable naïve (CD19^+^CD27^−^) or memory (CD19^+^CD27^+^) B cells that stained positive for CellROX Deep Red probe (CellROX^+^ cells) (**Figure 2A**).

ROS fold increase induced by each single stimulus related to the unstimulated sample was expressed as a ratio: (single stimulus % CellROX^+^ cells)/(unstimulated % CellROX^+^ cells).

*In vitro* basal cell death was evaluated as the percentage of SYTOX^+^ naïve (CD19^+^CD27^−^) or memory (CD19^+^CD27^+^) B cells in unstimulated 24-h cultures (**Supplementary Figure 1A**).

#### 2.3.4 Autophagy

For autophagy detection, the Guava Autophagy LC3 Antibody-based detection kit (Luminex, Austin, TX, USA) was performed following the manufacturer’s instructions. Anti-LC3-II-FITC detects lipidated LC3-II protein present in the autophagosome membrane. Briefly, 5 × 10^5^ harvested cells were stained with Live/Dead Aqua Dead Cell Stain (Invitrogen Molecular Probes) for 20 min at 4°C. Cells were washed with cold PBS and stained with anti-CD19-APC and anti-CD27-BV605 (both from BD Biosciences) for 20 min at 4°C. After surface staining, cells were washed with 1X Assay Buffer and stained with 1X Autophagy Reagent B for selective permeabilization. Cells were washed immediately and stained with 1X Anti-LC3-II-FITC for 30 min at 4°C and finally fixed with 2% formaldehyde solution (Merck, Darmstadt, Germany) for 10 min at RT.

**Figure 3:**
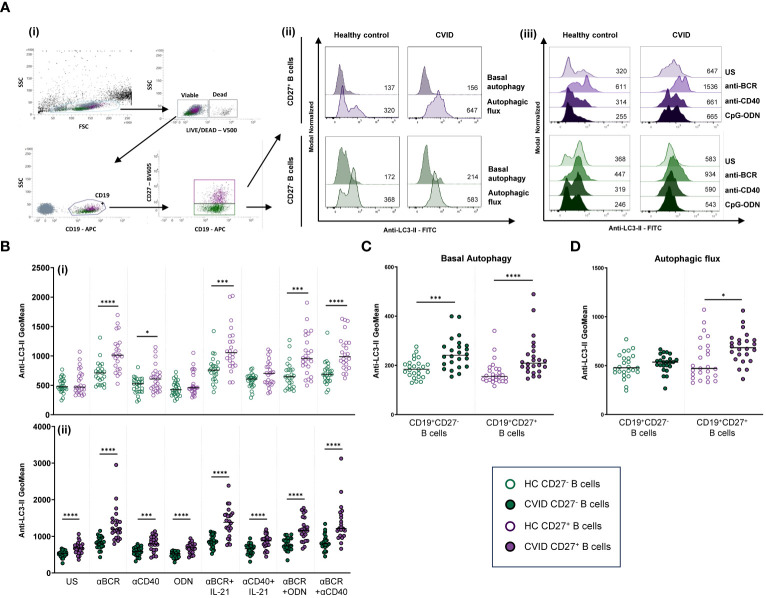
Autophagy in B-cell subpopulations from healthy controls and common variable immunodeficiency (CVID) patients. **(A)** Dot plot and histograms of autophagy levels in B-cell subpopulations from a representative control and CVID patient. (i) Gating strategy: viable B cells were selected based on forward and side scatter, non-expression of Live/Dead marker, and CD19^+^ expression. B-cell subpopulations were identified as naïve CD19^+^CD27^−^ (green color) and memory CD19^+^CD27^+^ (purple color). (ii) Anti-LC3-II mean fluorescence intensity (MFI) was used to evaluate autophagy levels. Histograms represent Anti-LC3-II MFI from a healthy control (left histograms) and a CVID patient (right histograms). Within individual plots, upper histogram refers to basal autophagy, and lower histogram refers to autophagic flux. Numbers in the histogram refer to Anti-LC3-II MFI. (iii) Representative histograms of autophagic flux without or with stimulation with anti-BCR, anti-CD40, or CpG-ODN in naïve CD19^+^CD27^−^ (green color) and memory CD19^+^CD27^+^ (purple color) from a healthy control (left histograms) and a CVID patient (right histograms). Numbers in the histogram refer to Anti-LC3-II MFI. **(B)** Comparison of autophagic flux between naïve CD19^+^CD27^−^ (green dots) and memory CD19^+^CD27^+^ (purple dots) B cells from (i) healthy controls and (ii) CVID patients after 24 h of culture without or with stimulation with anti-BCR, anti-CD40, CpG-ODN, anti-BCR+IL-21, anti-CD40+IL-21, anti-BCR+CpG-ODN, or anti-BCR+anti-CD40. **(C)** Basal autophagy in unstimulated naïve CD19^+^CD27^−^ (green color) and memory CD19^+^CD27^+^ (purple color) from healthy controls (empty dots) and CVID patients (filled dots). **(D)** Autophagic flux in unstimulated naïve CD19^+^CD27^−^ (green color) and memory CD19^+^CD27^+^ (purple color) from healthy controls (empty dots) and CVID patients (filled dots). **(B–D)** Each dot represents an individual; black horizontal lines illustrate the median of the group; Mann–Whitney test p*-*values: *p < 0.05; ***p < 0.001; ****p < 0.0001.

Geometric mean fluorescence intensity (MFI) of Anti-LC3-II-FITC was used to evaluate autophagy levels in previously gated naïve (CD19^+^CD27^−^) and memory (CD19^+^CD27^+^) viable (Live/Dead^−^) B cells. Basal autophagy refers to autophagy measured in 24-h unstimulated cultured cells. Autophagic flux refers to autophagy measured in 24-h unstimulated or stimulated cells in the presence of bafilomycin A1 (bafA1). Bafilomycin promotes the accumulation of autophagosomes by inhibiting autophagosome breakdown, thus favoring the detection of LC3-II (**Figure 3A**). Autophagic flux fold increase induced by each single stimulus related to the unstimulated sample was expressed as a ratio: (single stimulus Anti-LC3-II MFI)/(unstimulated Anti-LC3-II MFI).

### 2.4 Statistical analysis

Statistical analysis was performed using GraphPad Prism version 8.0 software. The Mann–Whitney test was used to compare differences between two independent data sets related to a single experimental condition (e.g., CD19^+^CD27^−^ *vs.* CD19^+^CD27^+^ unstimulated control B cells, or control *vs.* CVID single B-cell subpopulation stimulated with anti-BCR). The Wilcoxon test was used to compare differences between two paired groups of treatments (basal and post-stimulation conditions or each single stimulus with its combination with other stimuli). Correlation between variables was measured using Pearson’s correlation coefficient. A p-value less than 0.05 was considered statistically significant. Principal component analysis (PCA) was performed to evaluate relationships between the experimental variables studied and the patients’ B-cell deficiency according to the EUROclass classification. To this end, in the PCA, the categorical B-cell deficiency variable was converted to numerical data, as follows: healthy controls = 0, smB+ CVID patients = 1, and smB− CVID patients = 2. Variables were considered significantly loaded in the PCA when the loading value was above or below ±0.5.

## 3 Results

### 3.1 Cells with dysfunctional mitochondria in naïve and memory B cells from healthy controls and CVID patients

We evaluated the percentage of naïve (CD19^+^CD27^−^) and memory (CD19^+^CD27^+^) viable B cells with dysfunctional mitochondria from healthy controls and CVID patients. Representative data of a CVID patient and paired healthy control are depicted in **Figure 1A**ii.

Unstimulated CD19^+^CD27^−^ control B cells, in contrast to CVID B cells, exhibited a higher percentage of dysfunctional mitochondria than CD19^+^CD27^+^ control B cells (p < 0.01) (**Figures 1B**i, ii). Next, we compared the percentage of B-cell subpopulations with dysfunctional mitochondria between healthy controls and CVID patients. We detected higher basal percentages of CD19^+^CD27^−^ B cells with dysfunctional mitochondria in CVID patients compared to controls (p < 0.01) (**Figure 1C**).

**Figure 4:**
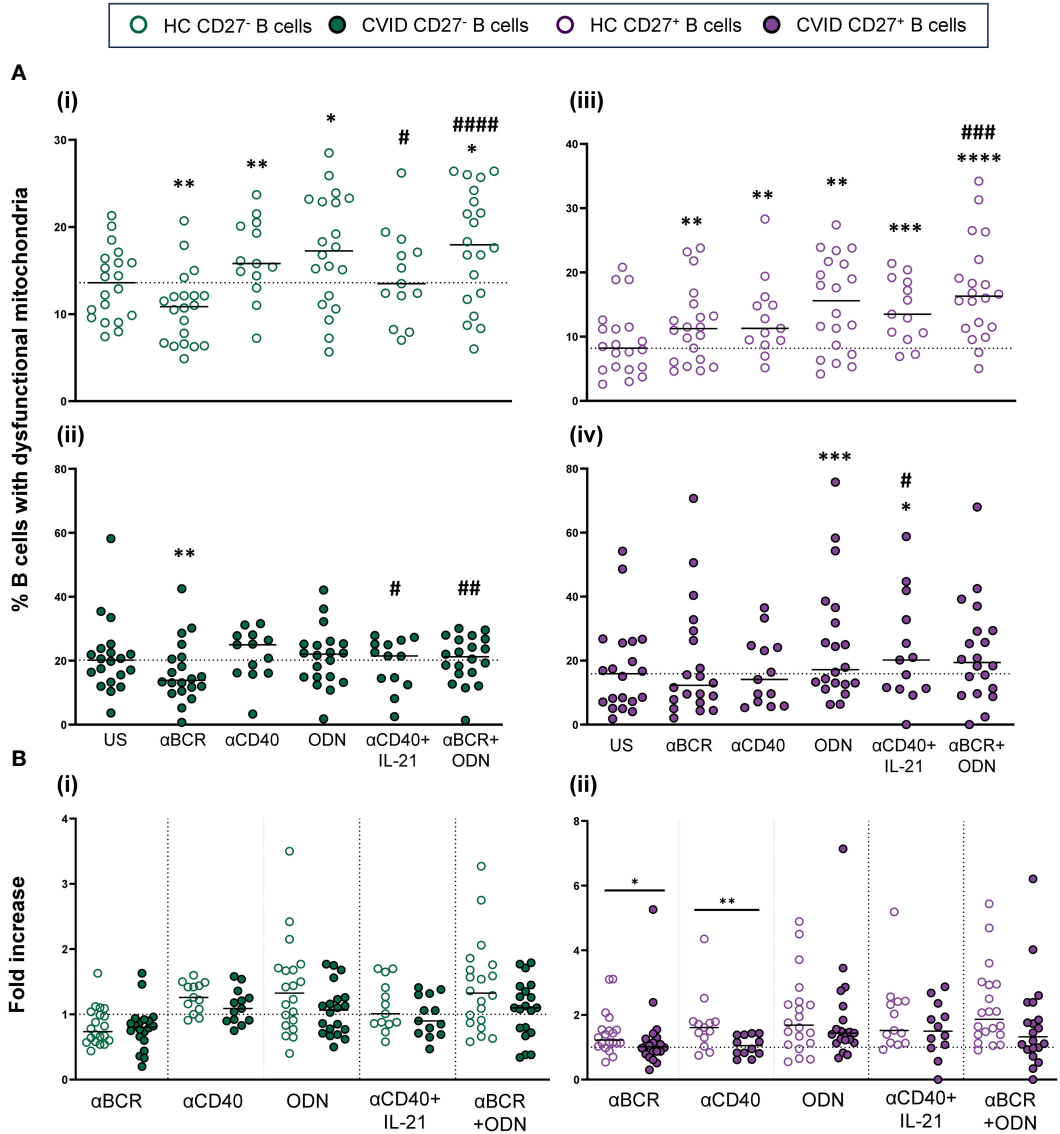
B-cell subpopulations with dysfunctional mitochondria in healthy controls and common variable immunodeficiency (CVID) patients after stimulation. **(A)** Percentages of naïve CD19^+^CD27^−^ (i) and (ii) and memory CD19^+^CD27^+^ (iii) and (iv) B cells with dysfunctional mitochondria in healthy controls (i) and (iii) and CVID patients (ii) and (iv) after 24 h of culture without or with stimulation with anti-BCR, anti-CD40, CpG-ODN, anti-CD40+IL-21, or anti-BCR+CpG-ODN. **(B)** Fold increase in the percentage of B-cell subpopulations with dysfunctional mitochondria induced by each single stimulus or combination, related to unstimulated sample in (i) naïve CD19^+^CD27^−^ and **(ii)** memory CD19^+^CD27^+^ B cell from healthy controls and CVID patients. Each dot represents an individual. Green dots (naïve CD19^+^CD27^−^) and purple dots (memory CD19^+^CD27^+^); empty dots (healthy controls) and filled dots (CVID patients). Black horizontal lines illustrate the median of the group. **(A)** Wilcoxon test p*-*values: *p < 0.05; **p < 0.01; ***p < 0.001; ^#^p < 0.05 (“*” refers to p*-*value of samples stimulated with a single stimulus compared to unstimulated sample; “#” refers to p*-*value of stimulus combinations compared to single stimulus). **(B)** Mann–Whitney test p*-*values: *p < 0.05, **p < 0.01.

The percentage of cells with dysfunctional mitochondria increased upon control B-cell activation through T-independent (CpG-ODN) and T-dependent (anti-CD40) stimuli (**Figures 4**i, iii). Specifically, CpG-ODN induced the strongest increase on both CD19^+^CD27^−^ and CD19^+^CD27^+^ control B cells (p < 0.05 and p < 0.01, respectively), followed by anti-CD40 (p < 0.01 and p < 0.01, respectively) (**Figures 4A**i, iii). Interestingly, B-cell activation by BCR triggering (anti-BCR) reduced the percentage of B cells with dysfunctional mitochondria in CD19^+^CD27^−^ (p < 0.01) while increasing their percentage in CD19^+^CD27^+^ control B cells (p < 0.01) (**Figures 4A**i, iii). The addition of IL-21 decreased the effect of anti-CD40 in CD19^+^CD27^−^ control B cells (p < 0.05) (**Figure 4A**i). The combination of anti-BCR with CpG-ODN increased the percentage of both CD19^+^CD27^−^ and CD19^+^CD27^+^ control B cells with dysfunctional mitochondria compared to anti-BCR alone (p < 0.0001 and p < 0.001, respectively) (**Figures 4A**i, iii).

Unlike what was observed in healthy controls, anti-CD40 and CpG-ODN did not increase the percentage of CD19^+^CD27^−^ CVID B cells with dysfunctional mitochondria (**Figures 4A**i, ii). After anti-BCR and anti-CD40 stimulation, there was a statistically lower fold increase only in the percentage of CD19^+^CD27^+^ CVID B cells with dysfunctional mitochondria compared to healthy controls (p < 0.05 and p < 0.01, respectively) (**Figures 4B**i, ii). Despite this, the final result was a generalized higher level of cells with dysfunctional mitochondria in both CVID subpopulations after stimulation (**Figures 4A**i–iv).

### 3.2 ROS levels and production in naïve and memory B cells from healthy controls and CVID patients

Next, we evaluated ROS production in naïve (CD19^+^CD27^−^) and memory (CD19^+^CD27^+^) viable B cells from healthy controls and CVID patients (**Figures 2A**i, ii).

Unstimulated CD19^+^CD27^+^ B cells from healthy controls displayed strongly higher ROS production than CD19^+^CD27^−^ B cells (p < 0.0001) (**Figure 2B**i). Unstimulated CD19^+^CD27^+^ B cells from CVID patients also displayed significantly higher ROS production than CD19^+^CD27^−^ B cells (p < 0.0001) (**Figure 2B**ii). Interestingly, unstimulated CD19^+^CD27^−^ CVID B cells presented higher ROS production than healthy controls (p < 0.0001) (**Figure 2C**).

**Figure 5:**
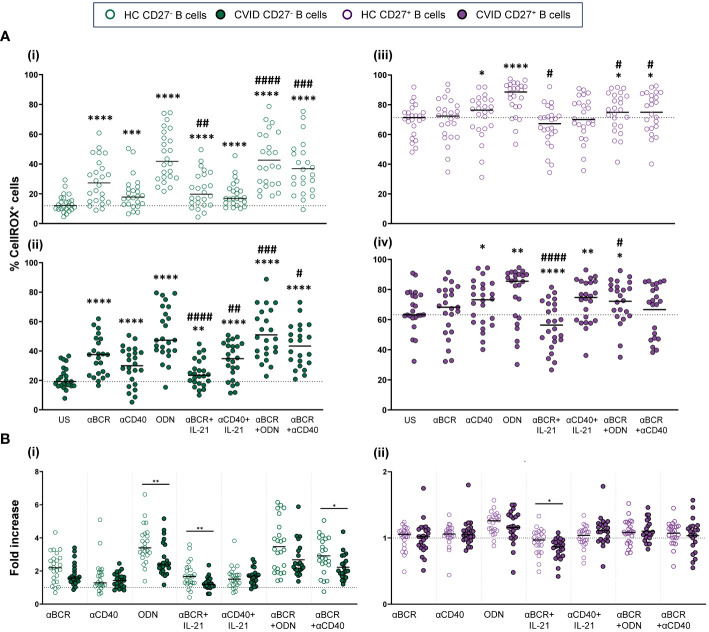
Reactive oxygen species (ROS) production in B cells from healthy controls and common variable immunodeficiency (CVID) patients after stimulation. **(A)** Percentages of CellROX^+^ naïve CD19^+^CD27^−^ (i) and (ii) and memory CD19^+^CD27^+^ (iii) and (iv) B cells in healthy controls (i) and (iii) and CVID patients (ii) and (iv) after 24 h of culture without or with stimulation with anti-BCR, anti-CD40, CpG-ODN, anti-BCR+IL-21, anti-CD40+IL-21, anti-BCR+CpG-ODN, or anti-BCR+anti-CD40. **(B)** Fold increase in the percentage of CellROX^+^ cells induced by each single stimulus or combinations, related to unstimulated sample in (i) naïve CD19^+^CD27^−^ and (ii) memory CD19^+^CD27^+^ B cells from healthy controls and CVID patients. Each dot represents an individual. Green dots (naïve CD19^+^CD27^−^) and purple dots (memory CD19^+^CD27^+^); empty dots (healthy controls) and filled dots (CVID patients). Black horizontal lines illustrate the median of the group. **(A)** Wilcoxon test p*-*values: *p < 0.05; **p < 0.01; ***p < 0.001; ****p < 0.0001; ^#^p < 0.05; ^##^p < 0.01; ^####^p < 0.0001 (“*” refers to p-value of samples stimulated with a single stimulus compared to unstimulated sample; “#” refers to p-value stimulus combinations compared to single stimulus). **(B)** Mann–Whitney test p*-*values: *p < 0.05, **p < 0.01.

The stimulation of CD19^+^CD27^−^ healthy control B cells with CpG-ODN, anti-BCR, and anti-CD40 alone increased ROS production (p < 0.0001, p < 0.0001, and p < 0.001, respectively), with CpG-ODN being the strongest inductor (**Figure 5A**i). The addition of IL-21 down-modulated ROS production induced by anti-BCR (p < 0.01) (**Figure 5A**i). Conversely, a synergic effect was observed when anti-BCR and anti-CD40 were combined compared to anti-BCR alone (p < 0.001) (**Figure 5A**i).

In CD19^+^CD27^+^ healthy control B cells, CpG-ODN and anti-CD40 stimulation significantly increased ROS production (p < 0.0001 and p < 0.05, respectively) (**Figure 5A**iii). This was not the case for anti-BCR stimulation. Like that observed in CD19^+^CD27^−^ B cells, IL-21 diminished ROS production induced by anti-BCR (p < 0.05) (**Figure 5A**iii). In any case, CD19^+^CD27^+^ B cells from healthy controls exhibited strongly higher ROS levels than CD19^+^CD27^−^ B cells with all stimuli and combinations used (**Figure 2B**i).

The behavior of CD19^+^CD27^−^ and CD19^+^CD27^+^ B cells regarding ROS production upon stimulation was similar between CVID patients and healthy controls (**Figures 5A**i–iv). However, CD19^+^CD27^−^ CVID B cells showed a lower ROS fold increase compared to healthy controls when stimulated with CpG-ODN, anti-BCR+IL-21, and anti-BCR+anti-CD40 (p < 0.01, p < 0.01, and p < 0.05, respectively) (**Figure 5B**i). Anti-BCR+IL-21 induced a significant decrease in ROS production by CD19^+^CD27^+^ CVID B cells (p < 0.0001) (**Figure 5A**iv), which resulted in a higher fold decrease compared to healthy controls (p < 0.05) (**Figure 5B**ii).

We found a negative correlation between percentages of dead cells and ROS levels (p < 0.001) in CVID CD19^+^CD27^+^ B cells (**Supplementary Figure 1C**ii).

### 3.3 Autophagy levels and autophagic flux in naïve and memory B cells from healthy controls and CVID patients

In naïve (CD19^+^CD27^−^) and memory (CD19^+^CD27^+^) B-cell subsets from healthy controls and CVID patients, we evaluated basal autophagy levels and autophagic flux as described in the Autophagy section of the Materials and Methods (**Figures 3A**i, ii).

Unstimulated CD19^+^CD27^−^ B cells from healthy controls displayed similar levels of autophagic flux to CD19^+^CD27^+^ control B cells (**Figure 3B**i). Unlike healthy controls, CD19^+^CD27^+^ from CVID patients exhibited higher autophagic flux compared to CD19^+^CD27^−^ CVID B cells (p < 0.0001) (**Figure 3B**ii).

Both unstimulated CD19^+^CD27^−^ and CD19^+^CD27^+^ B cells from CVID patients showed increased levels of basal autophagy compared to healthy control counterparts (p < 0.001 and p < 0.0001) (**Figure 3C**). Likewise, autophagic flux was higher in CD19^+^CD27^+^ CVID B cells compared to healthy controls (p < 0.05) (**Figure 3D**).

**Figure 6:**
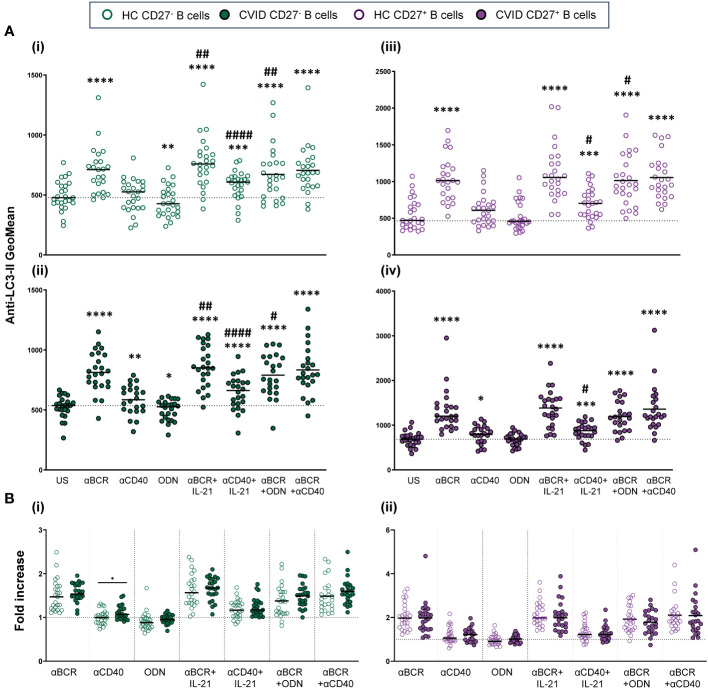
Autophagic flux in stimulated B-cell subpopulations from healthy controls and common variable immunodeficiency (CVID) patients. **(A)** Autophagic flux of naïve CD19^+^CD27^−^ (i) and (ii) and memory CD19^+^CD27^+^ (iii) and (iv) B cells in healthy controls (i) and (iii) and CVID patients (ii) and (iv) after 24 h of culture without or with stimulation with anti-BCR, anti-CD40, CpG-ODN, anti-BCR+IL-21, anti-CD40+IL-21, anti-BCR+CpG-ODN, or anti-BCR+anti-CD40. **(B)** Fold increase in autophagic flux induced by each single stimulus or combination, related to unstimulated sample in (i) naïve CD19^+^CD27^−^ and (ii) memory CD19^+^CD27^+^ B cells from healthy controls and CVID patients. Each dot represents an individual. Green dots (naïve CD19^+^CD27^−^) and purple color (memory CD19^+^CD27^+^); empty dots (healthy controls) and filled dots (CVID patients). Black horizontal lines illustrate the median of the group. **(A)** Wilcoxon test p*-*values: *p < 0.05; **p < 0.01; ***p < 0.001; ****p < 0.0001; ^#^p < 0.05; ^##^p < 0.01; ^####^p < 0.0001 (“*” refers to p-value of samples stimulated with a single stimulus compared to unstimulated sample; “#” refers to p-value stimulus combinations compared to single stimulus)). **(B)** Mann–Whitney test p*-*values: *p < 0.05.

Anti-BCR strongly increased autophagic flux in both CD19^+^CD27^−^ and CD19^+^CD27^+^ B cells from healthy controls (p < 0.0001 and p < 0.0001) (**Figures 6A**i, iii). The increase was more pronounced in CD19^+^CD27^+^ than CD19^+^CD27^−^ subpopulation (p < 0.0001) (**Figure 3B**i). In CD19^+^CD27^−^ B cells, the addition of IL-21 increased autophagic flux when combined with anti-BCR or anti-CD40, compared to those stimuli alone (p < 0.01 and p < 0.0001, respectively), whereas in CD19^+^CD27^+^ B cells, IL-21 only increased autophagic flux when combined with anti-CD40 (p < 0.05) (**Figures 6A**i, iii). Conversely, CpG-ODN alone reduced autophagic flux in CD19^+^CD27^−^ B cells (p < 0.01) (**Figure 6A**i). Likewise, CpG-ODN decreased autophagic flux induced by anti-BCR in both CD19^+^CD27^−^ and CD19^+^CD27^+^ B cells (p < 0.01 and p < 0.05, respectively) (**Figures 6A**i, iii).

The abovementioned results were similar in CVID patients regarding the induction of autophagic flux and the differential behavior of CD19^+^CD27^−^ and CD19^+^CD27^+^ control B cells in response to distinct stimuli (**Figures 6A**i–iv). However, when we compared the fold increase in autophagy between CVID patients and healthy control B cells, we found a significantly higher fold increase in autophagic flux in CD19^+^CD27^−^ CVID B cells stimulated with anti-CD40 (p < 0.05) (**Figure 6B**i). No differences were found for CD19^+^CD27^+^ CVID B cells (**Figure 6B**ii).

### 3.4 Principal component analysis of the experimental variables, *in vitro* cell death, and B-cell deficiency

Next, we aimed to explore, by PCA, the potential relationships between the studied experimental variables related to B-cell metabolism and *in vitro* basal cell death (expressed as a percentage of SYTOX^+^ cells) and the degree of B-cell deficiency in our cohort of CVID patients.

**Figure 7:**
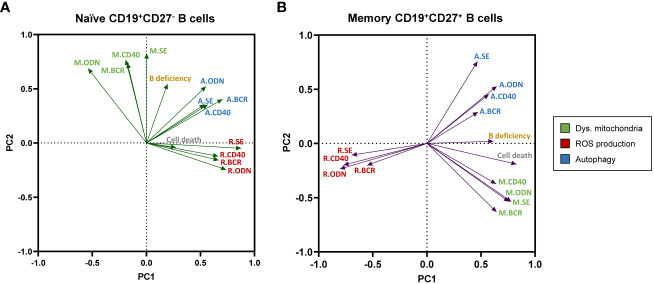
Principal component analysis (PCA) of experimental variables related to B-cell metabolism, “*in vitro*” B-cell death, and the degree of B-cell deficiency in our cohort of common variable immunodeficiency (CVID) patients. Loading plot of the first two principal components (PC1 and PC2) in naïve CD19^+^CD27^−^ **(A)** and memory CD19^+^CD27^+^ **(B)** B-cell subpopulations. M., R., and **(A)** refer to dysfunctional mitochondria (green), reactive oxygen species (ROS) production (red), and autophagy (blue) variables, respectively.

The PCA performed in CD19^+^CD27^−^ naïve B cells (**Figure 7A**) showed two principal components characterized by ROS production and autophagy [clustered in principal component 1 (PC1)] and mitochondrial dysfunction [clustered in principal component 2 (PC2)]. In this PCA, the only remarkable result observed was a moderate positive correlation between ROS production and autophagy, which formed medium acute angles (**Figure 7A**). As expected, the contribution of CD19^+^CD27^−^ naïve B cells *in vitro* basal cell death was irrelevant in the prediction model calculation (loading values of 0.273 and −0.042 in PC1 and PC2, respectively; **Figure 7A**). The B-cell deficiency degree showed a moderate positive correlation with mitochondrial dysfunction and autophagy; however, its loading value was very low in PC1 and slightly exceeded the proposed cut-off in PC2 (0.195 and 0.525, respectively, in **Figure 7A**).

Next, the PCA performed with data from CD19^+^CD27^+^ memory B cells (**Figure 7B**) yielded two principal components clearly shaped by ROS production and mitochondrial dysfunction in PC1 and autophagy in PC2. In this PCA, autophagy and mitochondrial function were apparently unrelated to them (nearly 90° angles formed) and, interestingly, showed a high negative correlation with ROS production (great obtuse angles formed). This was in accordance with the lower levels of ROS production and higher levels of autophagy previously demonstrated in CD19^+^CD27^+^ memory B cells from CVID patients (**Figures 2C**, **3C, D**, respectively). Moreover, CD19^+^CD27^+^ memory B cells *in vitro* basal cell death and B-cell deficiency degree clustered in PC1 (with ROS production and mitochondrial function) with high significant loading values (0.827 and 0.614, respectively, **Figure 7B**). Interestingly, the PCA in CD19^+^CD27^+^ memory B cells demonstrated a well-defined negative correlation between ROS production and either the *in vitro* basal cell death or the B-cell deficiency degree, given the nearly 180° angle that they formed (**Figure 7B**).

## 4 Discussion

ROS production, mitochondrial function, and autophagy are crucial for determining B-cell fate (31) and have been widely studied in mice. However, the interplay between these processes remains poorly investigated in human B cells. CVID patients present with deficient generation of memory B cells and antibody-secreting cells that are essential for the development of humoral immune responses.

The present study aimed to evaluate the interaction of mitochondrial function (mitochondrial membrane potential and mitochondrial mass), ROS production, and autophagy in naïve CD19^+^CD27^−^ and memory CD19^+^CD27^+^ B-cell subsets from healthy controls. Considering our previous results showing an imbalance in mitochondrial apoptosis regulation in memory B cells from CVID patients and an increased susceptibility to activation-induced apoptosis (27, 28), we also studied if alterations in these processes in CVID B-cell subpopulations could influence their fate and be the cause of their lack of differentiation in CVID patients.

Memory B cells differ from naïve B cells in important aspects including a lower threshold for activation, greater proliferative capacity, or survival period (32–35). Consistently, we found that healthy controls CD19^+^CD27^−^ and CD19^+^CD27^+^ B cells differentially depend on these studied processes for homeostasis. In unstimulated cell cultures, we found higher amounts of cells with dysfunctional mitochondria in CD19^+^CD27^−^ than in CD19^+^CD27^+^ B cells that, at the same time, displayed strongly lower ROS levels, despite showing similar autophagic flux.

ROS production plays a fundamental cellular dual role. At low levels, ROS act as second messengers essential in signal transduction. However, at high levels, ROS can cause organelle oxidative damage, particularly in mitochondria. ROS production increases when B cells are activated, playing a role as second messengers during B-cell activation and differentiation (31). In response to BCR stimulation, ROS production occurs in two waves: an early increase, within minutes upon stimulation, and a second wave of “mitochondrial” ROS production occurring at a later point (6–24 h) (36, 37). Wheeler et al. described that this late-phase ROS production is crucial for mouse spleen B-cell activation and survival. We evaluated this second and essential wave by studying healthy control B-cell subpopulation ROS production after 24-h stimulation. As expected, CD19^+^CD27^−^ B cells increased ROS production when stimulated with all single stimuli and their combinations, while CD19^+^CD27^+^ B cells, according to their lower threshold of activation, showed a moderate increase in ROS production (34, 35).

Mitochondria are the main source of ROS in the cell, and high ROS levels have been related to mitochondrial dysfunction (38). Surprisingly, there was a higher percentage of healthy CD19^+^CD27^−^ B cells with dysfunctional mitochondria than healthy CD19^+^CD27^+^, contrasting with their lower ROS levels. We found that different stimuli exerted a different effect on these processes. CpG-ODN was the stimulus that induced a higher increase of cells with dysfunctional mitochondria and ROS production in both healthy CD19^+^CD27^−^ and CD19^+^CD27^+^ B cells, supporting that mitochondria are the main source of ROS production in our model. We also observed that the effect of the stimuli was dependent on the cell type. Anti-BCR induced ROS production but decreased the percentage of cells with dysfunctional mitochondria in healthy control CD19^+^CD27^−^ cells, whereas it increased dysfunctional mitochondria, not increasing ROS production, in CD19^+^CD27^+^ B cells.

Autophagy is a cytoprotective pathway that protects cells from stressed conditions. However, disruption of autophagic mechanisms or excessive stress-induced autophagy, particularly by oxidative stress, may lead to “autophagic cell death” (39, 40). Memory B cells, as naïve B cells, are quiescent before antigen activation and their differentiation into antibody-secreting cells. Accordingly, resting healthy naïve and memory B cells had similar autophagy levels and autophagic flux. BCR stimulation and B-cell activation promote autophagy (16, 23). Consequently, anti-BCR and anti-BCR+IL-21 were the stimuli that induced higher autophagy levels in both CD19^+^CD27^−^ and CD19^+^CD27^+^ healthy B cells. CpG-ODN reduced autophagy in CD19^+^CD27^−^ and had no effect on CD19^+^CD27^+^ B cells, confirming the specific action of stimuli and the different cell type responses.

When CVID patients were studied, we found that unstimulated CD19^+^CD27^−^ CVID B cells exhibited a higher percentage of cells with dysfunctional mitochondria, lower ROS levels, but in a different way than healthy cells, and less autophagy compared to CD19^+^CD27^+^ CVID B cells. Moreover, dysfunctional mitochondria, ROS production, and autophagy were higher in both unstimulated and stimulated CD19^+^CD27^−^ CVID B cells than in their healthy counterparts, which was significant with certain stimuli. Concerning CD19^+^CD27^+^ CVID B cells, there was a lower ROS production and higher levels of dysfunctional mitochondria and autophagy than their healthy counterparts.

B cells from CVID patients displayed a ROS dysregulation compared to their healthy counterparts. Naïve B cells from CVID patients had higher basal ROS levels than controls, but interestingly, the ROS fold increase after stimulation was lower and never reached the same levels achieved by control B cells. Although ROS are required for B-cell activation and maturation, excessive ROS lead to oxidative stress (21, 22). Therefore, in CD19^+^CD27^−^ CVID B cells, the combination of high levels of basal ROS with their lower increase after stimulation suggests a ROS dysregulation that can result in dampened cell signaling. This could be detrimental to CVID naïve B-cell activation and differentiation to memory B cells.

Additionally, CVID patients’ CD19^+^CD27^+^ B-cell compartment had lower ROS basal levels and even a significant decrease in ROS production after anti-BCR+IL-21 stimulation, compared to healthy controls. In keeping with this, in a previous study, we demonstrated that CD19^+^CD27^+^ CVID B cells failed to upregulate anti-apoptotic Bcl-2 and Bcl-XL after anti-BCR+IL-21 stimulation (28). Interestingly, the dysfunction of ROS generation has been correlated with the reduction of memory B-cell compartment in chronic granulomatous disease (41). The decreased levels of ROS found in CD19^+^CD27^+^ B cells could be detrimental, leading to their premature death. We previously reported an increased CVID basal level of Bax and Bim that correlated with low viability and high Caspase-3 activation only in CVID memory CD19^+^CD27^+^ B cells (28). In keeping with these, we found a negative correlation between ROS production and *in vitro* cellular death in unstimulated CD19^+^CD27^+^ CVID B cells that were not found in their healthy counterpart (**Supplementary Figures 1C**i, ii), indicating that dysregulation of ROS balance contributes to the fact that peripheral CVID memory B cells are prompted to die from apoptosis.

As previously mentioned, autophagy plays a crucial role in B-cell activation, and BCR stimulation promotes autophagy and triggers apoptosis if B cells are not co-stimulated with a second signal (16, 23). CVID B cells exhibited higher basal autophagy levels in both CD19^+^CD27^−^ and CD19^+^CD27^+^ B subpopulations than their healthy counterparts; moreover, CD19^+^CD27^+^ CVID B cells also showed higher autophagic flux. CVID B cells also exhibited higher autophagic flux levels than their healthy counterparts when stimulated with most of the stimuli. Hence, excessive autophagy reported in CVID memory B cells could reflect the loss of cellular homeostasis and be detrimental to their survival.

PCA allowed us to link the studied metabolic experimental variables to *in vitro* B-cell death and B-cell deficiency. The analysis showed a different interaction of the evaluated metabolic variables in naïve CD19^+^CD27^−^ and memory CD19^+^CD27^+^ B cells. In naïve B cells, ROS production and autophagy clustered in the same component with a slightly positive correlation. Moreover, *in vitro* naïve cell death and B-cell deficiency degree variables had no relevance in the PCA model. Even though naïve CVID B cells presented an alteration in ROS levels and production that could condition their differentiation, the PCA suggested that the production of ROS and the autophagy process compensate each other, which leads to cell survival. In fact, there was no higher *in vitro* cell death in CVID naïve B cells compared to healthy controls (28) (**Supplementary Figure 1B**i).

However, we found that increased CVID memory B-cell death negatively correlated with ROS production (**Supplementary Figures 1B**ii, **C**ii). In PCA of memory B cells, ROS production, and autophagy were clearly “opposed” (they were placed in different components and formed open angles). Moreover, *in vitro* memory B-cell death and B-cell deficiency degree variables were relevant in the model (high loading), positively correlating with autophagy and negatively correlating with ROS production. The analysis indicates that, in this case, ROS level reduction occurs at the expense of uncontrolled autophagy that, finally, induces cell death.

As a limitation of this work, we should mention the lack of whole-exome/genome sequencing data of our cohort of patients. It would be interesting to extend the study to a larger patient sample and try to correlate these findings with possible underlying mutations.

In summary, we have found that naïve and memory healthy B cells differentially depend on mitochondrial function, ROS production, and autophagy for their integrity and function. CVID B-cell subpopulations show a loss of cellular homeostasis. An excessive autophagic flux, higher levels of dysfunctional mitochondria, and an alteration in ROS levels could affect the differentiation of CVID naïve into memory B cells while conditioning a higher susceptibility of CVID memory B cells to premature death. The final consequence is a failure in the generation of a functional B-cell compartment in CVID patients.

## Data availability statement

The raw data supporting the conclusions of this article will be made available by the authors, without undue reservation.

## Ethics statement

The studies involving humans were approved by Balearic Islands Clinical Research Ethics Committee (approval with reference: IB 4322/20). The studies were conducted in accordance with the local legislation and institutional requirements. The participants provided their written informed consent to participate in this study.

## Author contributions

MB-R: Investigation, Methodology, Visualization, Writing – original draft, Writing – review & editing, Data curation, Formal analysis, Software. VC: Investigation, Methodology, Supervision, Validation, Visualization, Writing – review & editing, Formal analysis. AC: Investigation, Formal analysis, Visualization, Writing – review & editing. AL-G: Investigation, Writing – review & editing, Methodology. JP: Project administration, Writing – review & editing, Resources. JF: Writing – review & editing, Conceptualization, Funding acquisition, Project administration, Supervision, Visualization, Writing – original draft.
