# Supplementary material for: Dysfunctional mitochondria, disrupted levels of reactive oxygen species, and autophagy in B cells from common variable immunodeficiency patients
Source: Front Immunol. 2024 Mar 26;15:1362995. doi: 10.3389/fimmu.2024.1362995 (PMC11002182; doi:10.3389/fimmu.2024.1362995)
Supplement: Supplementary file 1 [file DataSheet_1.zip › Figure S1.pptx]

## Slide 1
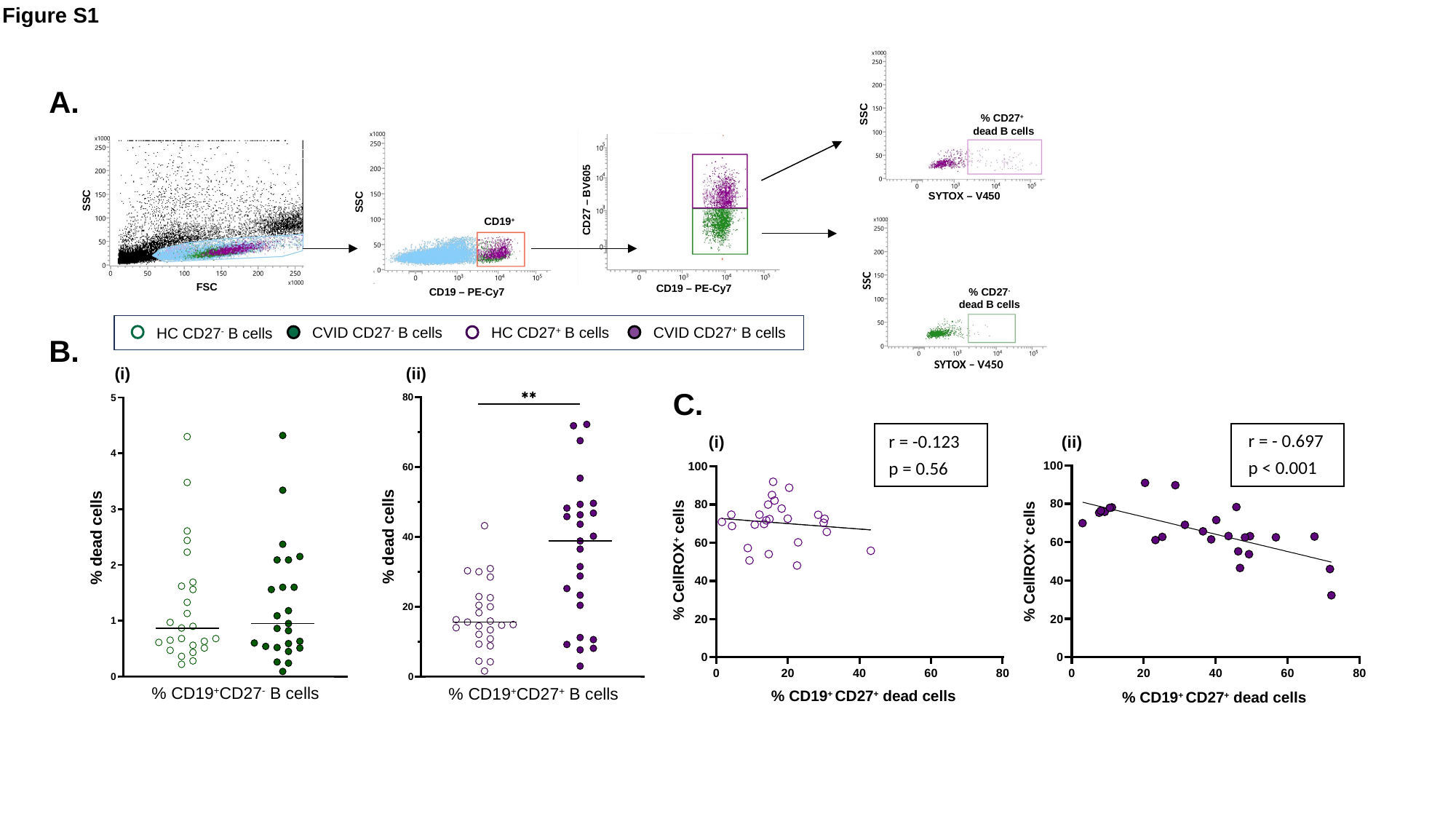

Figure S1
SSC
% CD27+
dead B cells
SYTOX – V450
A.
SSC
CD19+
CD19 – PE-Cy7
SSC
FSC
CD27 – BV605
CD19 – PE-Cy7
SSC
% CD27- dead B cells
SYTOX – V450
CVID CD27- B cells
HC CD27- B cells
CVID CD27+ B cells
HC CD27+ B cells
B.
(i)
(ii)
C.
% CD19+CD27- B cells
% dead cells
% dead cells
% CD19+CD27+ B cells
r = - 0.697
p < 0.001
% CellROX+ cells
% CD19+ CD27+ dead cells
(ii)
r = -0.123
p = 0.56
(i)
% CellROX+ cells
% CD19+ CD27+ dead cells

## Slide 2
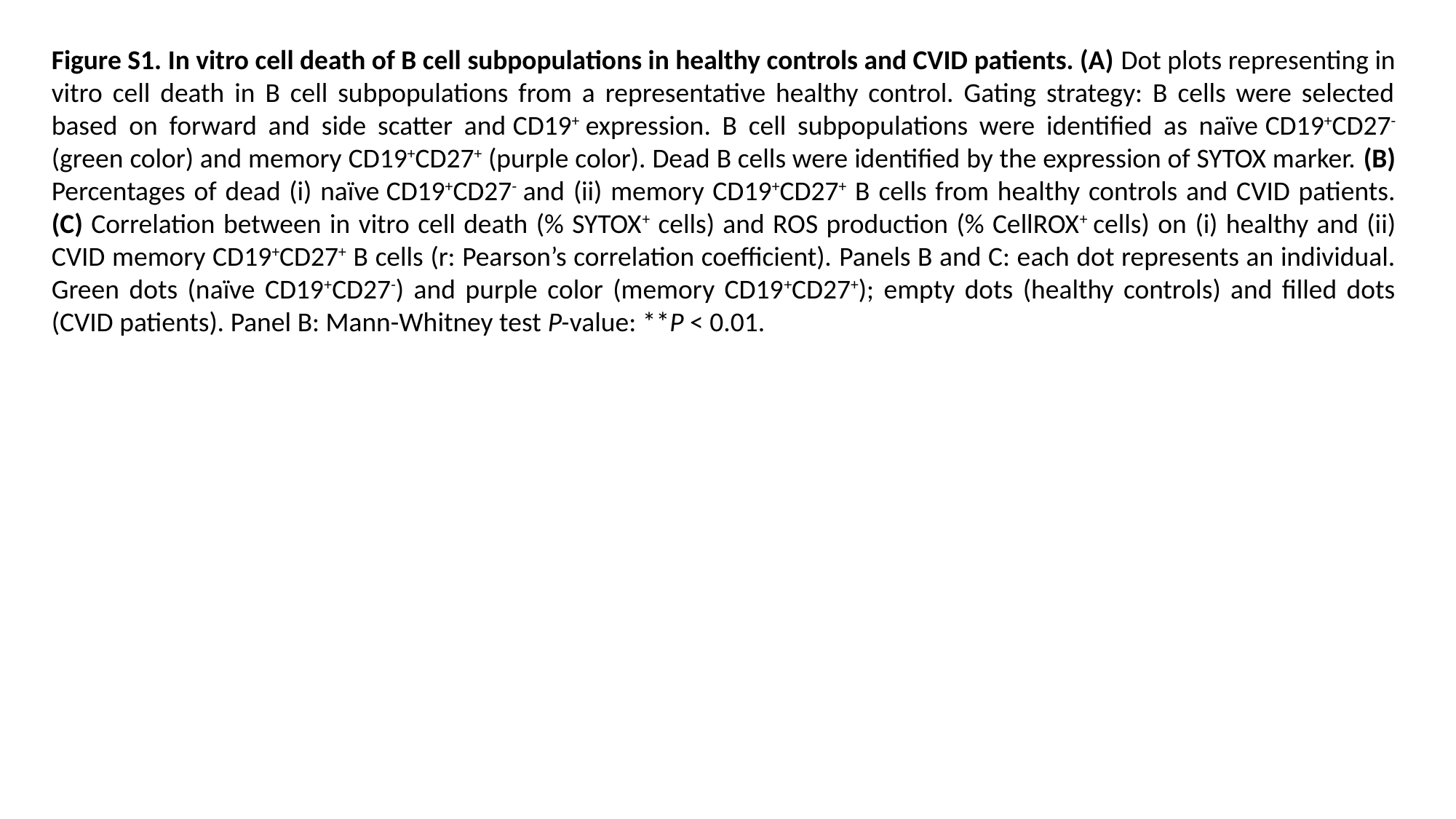

Figure S1. In vitro cell death of B cell subpopulations in healthy controls and CVID patients. (A) Dot plots representing in vitro cell death in B cell subpopulations from a representative healthy control. Gating strategy: B cells were selected based on forward and side scatter and CD19+ expression. B cell subpopulations were identified as naïve CD19+CD27-(green color) and memory CD19+CD27+ (purple color). Dead B cells were identified by the expression of SYTOX marker. (B) Percentages of dead (i) naïve CD19+CD27- and (ii) memory CD19+CD27+ B cells from healthy controls and CVID patients. (C) Correlation between in vitro cell death (% SYTOX+ cells) and ROS production (% CellROX+ cells) on (i) healthy and (ii) CVID memory CD19+CD27+ B cells (r: Pearson’s correlation coefficient). Panels B and C: each dot represents an individual. Green dots (naïve CD19+CD27-) and purple color (memory CD19+CD27+); empty dots (healthy controls) and filled dots (CVID patients). Panel B: Mann-Whitney test P-value: **P < 0.01.
